# Precision genomic profiling in Gaucher disease: insights from atypical presentations

**DOI:** 10.3389/fgene.2025.1553036

**Published:** 2025-11-07

**Authors:** Armaan Saith, Noor Ul Ain, Jiapeng Ruan, Maniya Kasaiyan, Dhanpat Jain, Gary Israel, Sameet Mehta, Nigel S. Bamford, Shiny Nair, Pramod K. Mistry

**Affiliations:** 1 Department of Internal Medicine, Yale School of Medicine, New Haven, CT, United States; 2 Department of Pediatrics, Yale School of Medicine, New Haven, CT, United States; 3 Department of Pathology, Yale School of Medicine, New Haven, CT, United States; 4 Department of Radiology, Yale School of Medicine, New Haven, CT, United States; 5 Department of Internal Medicine, Yale Center for Genome Analysis, New Haven, CT, United States; 6 Department of Neurology, Yale School of Medicine, New Haven, CT, United States; 7 Department of Cellular and Molecular Physiology, Yale University, New Haven, CT, United States

**Keywords:** Gaucher disease, modifier genes, complex phenotypes, longitudinal deep phenotyping, whole-exome sequencing

## Abstract

**Background:**

Gaucher disease (GD) is characterized by significant phenotypic heterogeneity, even among patients with identical *GBA1* genotypes, suggesting the role of genetic and/or epigenetic modifiers. The enzymatic defect and pathological accumulation of glucosylceramide (GlcCer) lead to chronic metabolic inflammation, providing ample opportunities for interaction with other biological pathways to influence disease expression. Herein, we developed a model of precision medicine in this prototype single-gene disorder.

**Methods:**

This study leveraged a well-characterized, longitudinally followed cohort of GD patients from a major tertiary care center, integrating whole-exome sequencing (WES) with detailed clinical information. We applied a precision medicine framework centered on four components—clinical reasoning, deep phenotyping, genomic integration, and individualized therapy—to a subset of patients (n = 17) who presented with complex phenotypes deviating from the classical GD presentation and/or were *a priori* suspected of harboring a second genetic disorder.

**Results:**

Of 275 patients, 17 (6.2%) presented with atypical phenotypes not fully explained by GD. WES revealed additional genetic diagnoses, including hereditary hemochromatosis-associated variants (n = 5), familial Mediterranean fever (n = 4), homozygous *MSH6* mutation-associated hereditary cancer predisposition (n = 2), and autosomal dominant polycystic kidney disease (ADPKD) (n = 2).

**Conclusion:**

The presence of concurrent genetic disorders in a subset of GD patients has the potential to modify clinical presentation, impact disease trajectory, and introduce additional complexities in clinical management. This study contributes to advancing precision medicine strategies that aim to optimize patient outcomes. Future research into genetic and epigenetic modifiers of GD will further refine this framework and enhance individualized therapeutic approaches.

## 1 Introduction

Gaucher disease (GD) is a prototype lysosomal storage disorder caused by biallelic mutations in the *GBA1* gene. These mutations lead to a deficiency of the lysosomal enzyme acid β-glucosidase (Grabowski et al., 2021). The enzymatic defect results in the accumulation of bioactive lipids, glucosylceramide (GlcCer), and glucosylsphingosine (GlcSph). Consequently, there is a generalized dysfunction of the lysosomal system, immune activation, and metabolic inflammation. The hallmark of GD pathology is the multisystemic buildup of lipid-laden macrophages, known as Gaucher cells. GD is broadly classified into three phenotypes: type 1 (GD1) (non-neuronopathic, lacking childhood-onset neurodegenerative disease) and the childhood-onset neuronopathic forms, type 2 (GD2) (acute, infantile onset) and type 3 (GD3) (chronic) (Mistry et al., 2011). While some genotype–phenotype correlations exist—such as the association of the p.Leu483Pro homozygous mutation with neuronopathic disease (nGD) and the p.Asn409Ser mutation with type 1 GD—phenotypic variability remains striking (Grabowski et al., 2019). Even among individuals with identical *GBA1* genotypes or among affected siblings, there can be profound differences in disease severity and presentation, challenging the traditional genotype–phenotype model (Sidransky, 2012).

While GD is a monogenic disorder, this variability suggests that additional factors, including genetic, epigenetic, or environmental modifiers, may play significant roles in shaping the clinical manifestations of the disease. By modulating penetrance, expressivity, and pleiotropy, such modifiers can intricately adjust cellular and organismic responses to *GBA1* mutations, thereby sculpting the clinical landscape of GD beyond the confines of traditional genotype–phenotype correlations. These modifiers may influence critical pathways, such as immune regulation, inflammation, and lysosomal function, further complicating disease expression. However, genotype–phenotype studies in GD have been limited by its rarity and extreme heterogeneity, making it difficult to draw robust conclusions from small cohorts. Some researchers have approached GD as a phenotypic continuum, ranging from asymptomatic GD1 to severe GD2 (Sidransky, 2004). Genome-wide association studies (GWAS) and candidate gene studies have offered insights, but much remains unexplained (Zhang et al., 2012; Velayati et al., 2011; Lo et al., 2011).

We posit that a phenotype-first approach has broad utility in GD for investigating the impact of genomic background and modifier genes underlying the phenotypic heterogeneity observed among patients with identical *GBA1* mutations. This approach not only helps elucidate disease variability but also contributes to the development of a precision medicine model for GD. Notably, significant advances have been made using this strategy in patients with GD who develop Parkinson’s disease (Blauwendraat et al., 2023).

Our long-term goal has been to understand the genomic basis of phenotypic diversity in GD (Zhang et al., 2012). To achieve this, our cohort underwent deep phenotyping and whole-exome sequencing (WES) as part of a systematic investigation into disease variability. Here, we focused on a subset of 17 patients who deviated from the classical GD phenotype due to multiple molecular diagnoses and/or were *a priori* suspected of harboring a second genetic disorder based on family history. Our objective was to evaluate the utility of WES in Gaucher patients with atypical presentations to investigate the genomic basis of phenotypic variability, and to develop a precision medicine framework for individualized patient care. Leveraging this approach, we examined the WES data from this subset to correlate genetic findings with their respective phenotypes. The cases described highlight key pillars of precision medicine as applied to a single-gene disease order, namely, deep phenotyping, longitudinal natural history, clinical reasoning, genomic data, and individualized therapy for holistic disease management.

## 2 Materials and methods

### 2.1 Study design and cohort

The Yale Gaucher Disease Center is a destination center for GD patients who undergo comprehensive evaluations 1–2 times per year. Our cohort (n = 275) has been followed for up to 26 years (range: 7 months to 26 years), with systematic collection of clinical, genetic (WES), and laboratory data. All patients were confirmed to have GD based on low acid β-glucosidase activity in peripheral blood leucocytes and biallelic *GBA1* mutations. For precise *GBA1* genotyping, we employed a combination of PacBio long-read sequencing, WES, and Sanger sequencing to ensure accurate variant detection (Drelichman et al., 2021).

**FIGURE 1:**
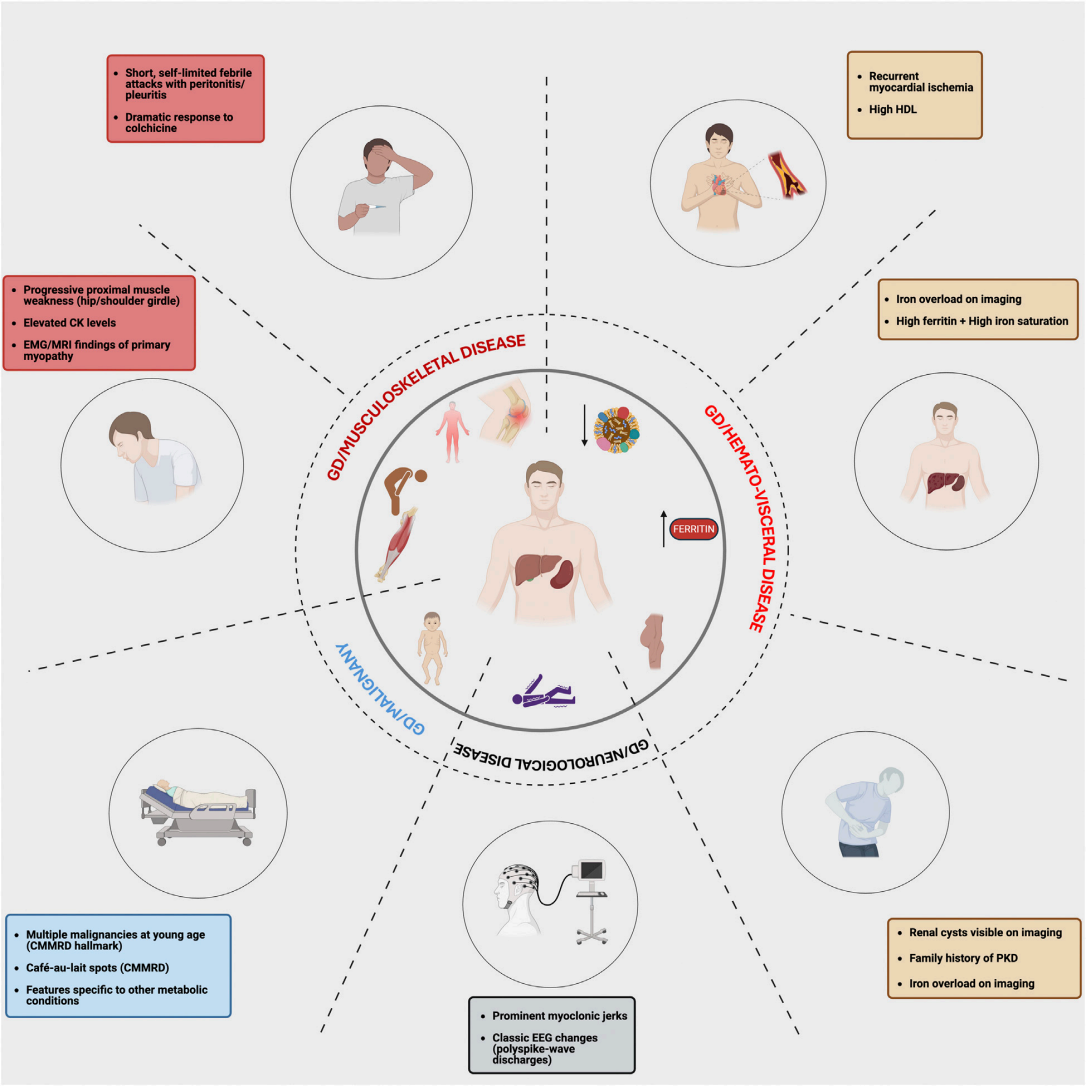
Atypical features in Gaucher disease that prompt evaluation for multiple molecular diagnoses. This figure maps organ system findings in Gaucher disease that should trigger genomic review. Recognizing these phenotypic red flags in distinct clinical domains may prompt clinicians to move beyond a “single gene-single disease” model and consider the presence of a second genetic diagnosis or a potential modifier.

We employ a phenotype-first approach to investigate the genetic basis of phenotypic variability in GD. Here, we focused on a subset of 17 patients who exhibited unusual phenotypes in GD. The concept for our study is depicted in Figure 1, which illustrates the spectrum of atypical phenotypic features observed in our cohort that signaled the presence of additional genetic diagnoses beyond Gaucher disease. By mapping clinical “red flags” across musculoskeletal, hematologic/visceral, neurologic, oncologic, and cardiovascular domains, the figure highlights how seemingly incongruent findings such as recurrent febrile serositis, florid proximal myopathy, iron overload with high ferritin and saturation, early onset malignancies, or renal cystic disease should prompt clinicians to move beyond a monogenic framework and pursue genomic evaluation. This approach highlights the central premise of our study: that GD, despite its single-gene basis, can present with blended or expanded phenotypes due to concurrent genetic disorders or modifier variants. Recognizing these phenotypic outliers is essential for precision medicine as it allows tailoring of diagnostic work-up and therapy to the full spectrum of a patient’s molecular diagnoses, thereby refining prognosis and management. Supplementary Table S1 lists each of the 17 patients, their *GBA1* genotype, age at GD diagnosis, therapy (ERT or substrate reduction therapy (SRT)), second diagnosis, and age of second diagnosis.

### 2.2 Longitudinal deep phenotyping

Deep phenotyping included assessments of organ volumes, bone disease indicators, and extensive laboratory testing, including GD biomarkers. This longitudinal approach provided a robust dataset to capture the natural history of GD across various phenotypes.

### 2.3 Whole-exome sequencing (WES) and analysis

WES was performed as described previously, with additional modifications outlined in Supplementary Materials S1 (Lo et al., 2012). We integrated clinicopathological correlations with genetic data to investigate potential interactions between GD and other disease pathways. Variants in genes encoding proteins involved in key cellular pathways were analyzed and classified according to American College of Medical Genetics (ACMG) guidelines for pathogenicity. This integrative approach facilitated structured clinical reasoning to identify potential correlations between specific genetic variations and the complex phenotypic presentations observed in this subset of patients.

### 2.4 Ethical considerations

This study adhered to all relevant ethical guidelines and was approved by the Institutional Review Board at Yale University. All participants provided informed consent and written informed consent, including explicit permission for the publication of de-identified genetic data.

## 3 Results

We focused on 17 patients, including 16 with GD1 and 1 with GD3, who exhibited expanded phenotypes that deviated from the typical presentation of GD, prompting further evaluation for potential concurrent genetic disorders (Tables 1, 2) (Figure 1). The classical phenotypic spectrum of GD1 includes varying degrees of hepatosplenomegaly, cytopenia, and skeletal disease. However, the selected patients displayed atypical manifestations, including symptoms that were either unusually severe, unexplained by known GD pathology, or suggestive of additional systemic involvement. These atypical features warranted WES to investigate potential genetic modifiers or coexisting genetic conditions.

**TABLE 1:** Our cohort of 17 patients harboring another rare disorder in addition to Gaucher disease.

Concomitant genetic disorder	OMIM code	ORPHA code	Number of cases
Familial Mediterranean fever	249,100	342	4
Metachromatic leukodystrophy	250,100	512	1
Fibromuscular dysplasia	135,580	698,012	1
Constitutional mismatch repair deficiency syndrome	619,097	252,202	2
Myoclonic epilepsy	254,770	307	1
Brugada syndrome	611,876	130	1
Autosomal dominant polycystic kidney disease	173,900	730	2
Iron dysregulation	235,200, 606,069	4,565,508, 647,834	5

**TABLE 2:** Demographic details of our cohort of patients with complex phenotype GD. This table highlights the demographic and clinical characteristics of GD patients at our center, comparing those with complex phenotypes (N = 17) to our total cohort (N = 275).

Demographic	Complex phenotype GD (N = 17)	Total cohort (N = 275)
Age (years) [Mean (Range)]	43.75 (12–74)	46.4 (1–94)
Gender (female) N (%)	10 (58.8)	142 (51.6)
*GBA1* genotype (p.Asn409Ser/p.Asn409Ser) N (%)	11 (64.7)	151 (54.9)
Splenectomy N (%)	1 (5.9)	15 (5.4)
ERT N (%)	8 (47.1)	133 (48.4)
SRT N (%)	9 (52.9)	107 (38.9)
Untreated N (%)	0 (0)	39 (14.2)

GD, Gaucher disease; ERT, Enzyme replacement therapy; SRT, Substrate reduction therapy.

The identified cases broadly fell into six categories:

1. Unusual inflammatory symptoms due to concurrent familial Mediterranean fever (FMF) in GD1 (n = 4).
2. Myopathy, unexpectedly severe osteoporosis in childhood in a patient homozygous for the p.Asn409Ser mutation, and debilitating chronic fatigue in GD1.
3. Complex phenotypes arising from another rare genetic disease (n = 5, GD1).
4. Autosomal dominant polycystic kidney disease (ADPKD) (n = 2, GD1).
5. Myoclonic epilepsy (n = 1, GD3).
6. Hyperferritinemia and iron overload (n = 5, GD1).

WES was analyzed to identify candidate genes, and the selected variants were annotated using ClinVar, sorting intolerant from tolerant (SIFT), minor allele frequency (MAF), and PolyPhen. Given that approximately 90% of our cohort is of Ashkenazi Jewish ancestry, allele frequencies were specifically examined in our entire cohort and in the broader population datasets in the public domain (Table 3).

**TABLE 3:** Genetic variants detected in our cohort of 17 patients. This table depicts the genetic variants detected in 17 patients with GD in our cohort. For each variant, we report the associated disease, affected gene, variant designation (SNP/coding change), variant type, zygosity status, amino acid change, SIFT prediction score, MAF in the general population, AJ-specific MAF, PolyPhen predicted functional impact, Yale GD ratio, ACMG classification, and rare exome variant ensemble learner (REVEL) score. SIFT scores range from 0 to 1, with scores ≤0.05 considered deleterious. PolyPhen and REVEL scores also range from 0 to 1, with higher scores indicating greater functional impact. The Yale GD ratio represents the relative frequency of variants in these 17 patients compared to our entire cohort of GD patients at Yale. AJ-MAF refers to the minor allele frequency in the Ashkenazi Jewish population.

Disease	Patient	Gene	Variant	Amino acid change	Type	Zygosity	SIFT	MAF	AJ-MAF*	PolyPhen	Yale GD ratio	ACMG classification	REVEL score
Familial Mediterranean fever	Patient 1	MEFV	rs1231123	p.Asp424Lys	Coding	Heterozygous	Tolerated (0.34)	0.3744	0.4184	Possibly damaging (0.631)	0.63	Benign	0.009
	Patient 2	MEFV	rs28940579	p.Val726Ala	Coding	Heterozygous	Tolerated (1)	0.002167	0.0421	Benign (0.06)	0.07	Pathogenic/likely pathogenic	0.35
	Patient 3	MEFV	rs28940579	p.Val726Ala	Coding	Heterozygous	Tolerated (1)	0.002167	0.0421	Benign (0.06)	0.07	Pathogenic/likely pathogenic	0.35
	Patient 4	MEFV	rs1231123	p.Asp424Lys	Coding	Heterozygous	Tolerated (0.34)	0.3744	0.4184	Possibly damaging (0.631)	0.63	Benign	0.009
Nemaline myopathy	Patient S5	NEB	rs750810441	p.Asp5398Asn	Coding	Heterozygous	NA	0.002193	0.0115	Unknown (0)	0.01	Likely benign**	0.071
	Patient S6	NEB	rs201825451	p.Asp8345 =	Coding	Heterozygous	NA	0.00157	0.017	NA	0.03	Conflicting classifications (Uncertain significance/benign/Likely benign)***	0.014
Hypophosphatasia	Patient S6	ALPL	rs148405563	p.Thr273Met	Coding	Heterozygous	Deleterious (0.04)	0.001675	0.0357	Benign (0.033)	0.06	Conflicting classifications (Uncertain significance/benign/likely benign)***	0.325
Metachromatic leukodystrophy	Patient 5	ARSA	rs867538940	p.Arg60Trp	Coding	Homozygous	Deleterious (0.03)	5.82E-06	0	Probably damaging (0.989)	0.01	Pathogenic/likely pathogenic	0.877
Fibromuscular dysplasia	Patient 6	YY1AP1	rs41264945	p.Gln242	Coding	Heterozygous	Deleterious (0)	0.03638	0.0308	Probably damaging (0.999)	0.04	NA	0.138
Brugada syndrome	Patient 7	CACNB2	rs875989812	NA	Splice acceptor	Heterozygous	NA	0.0006187	0.0026	NA	0.007	Uncertain significance	NA
Constitutional mismatch repair deficiency	Patient 8	MSH6	c.3822dupA	E1276X	Frame-shift	Homozygous	Deleterious (0)	0	0	Probably damaging (0.999)	0.008	Pathogenic	NA
	Patient 9	MSH6	c.3822dupA	E1276X	Frame-shift	Homozygous	Deleterious (0)	0	0	Probably damaging (0.999)	0.008	Pathogenic	NA
ADPKD	Patient 10	PKD1	rs138575342	p.Pro694Leu	Coding	Heterozygous	Deleterious (0)	0.02435	0	Probably damaging (0.993)	0.037	Uncertain significance	0.258
	Patient 11	PKD1	rs1282668884	p.Arg4150Cys	Coding	Heterozygous	Deleterious (0)	NA	0	Probably damaging (1)	0.037	Conflicting classifications (Pathogenic/uncertain significance)	0.676
Myoclonic epilepsy	Patient 12	EFHC1	rs1570624	p.Arg294His	Coding	Heterozygous	Deleterious (0)	0.01005	0.0043	Probably damaging (0.999)	0.02	Benign	0.668
Iron dysregulation	Patient 13	HFE	rs1799945	p.His63Asp	Coding	Homozygous	Tolerated (0.09)	0.1092	0.1208	Possibly damaging (0.704)	0.22	Conflicting classifications (Pathogenic/likely pathogenic/uncertain significance)	0.254
		SLC40A1	rs2304704	p.Val221 =	Splice site	Homozygous	NA	0.6286	0.7137	NA	0.89	Benign	NA
		SLC40A1	rs1439816	NA	Non-coding	Homozygous	NA	0.7967	0.8192	NA	0.96	Benign	NA
	Patient 14	HFE	rs1800562	p.Cys282Tyr	Coding	Heterozygous	Deleterious (0)	0.03321	0.0138	Possibly damaging (0.509)	0.06	Pathogenic	0.872
		SLC40A1	rs2304704	p.Val221 =	Splice site	Homozygous	NA	0.6286	0.7137	NA	0.89	Benign	NA
		SLC40A1	rs1439816	NA	Non-coding	Homozygous	NA	0.7967	0.8192	NA	0.96	Benign	NA
	Patient 15	HFE	rs1799945	p.His63Asp	Coding	Heterozygous	Tolerated (0.09)	0.1092	0.1208	Possibly damaging (0.704)	0.22	Conflicting classifications (Pathogenic/likely pathogenic/uncertain significance)	0.254
		SLC40A1	rs1439816	NA	Non-coding	Heterozygous	NA	0.7967	0.8192	NA	0.96	Benign	NA
		SLC40A1	rs11568351	NA	Non-coding	Heterozygous	NA	0.168	0.1275	NA	0.28	Benign	0.050
		SLC40A1	rs13008848	NA	Non-coding	Heterozygous	NA	0.1655	0.1252	NA	0.27	Benign	0.364
	Patient 16	HFE	rs1799945	p.His63Asp	Coding	Heterozygous	Tolerated (0.09)	0.1092	0.1208	Possibly damaging (0.704)	0.22	Conflicting classifications (Pathogenic/likely pathogenic/uncertain significance)	0.254
		SLC40A1	rs2304704	p.Val221 =	Splice site	Homozygous	NA	0.6286	0.7137	NA	0.89	Benign	NA
		SLC40A1	rs1439816	NA	Non-coding	Homozygous	NA	0.7967	0.8192	NA	0.96	Benign	NA
	Patient 17	SLC40A1	rs2304704	p.Val221 =	Splice site	Homozygous	NA	0.6286	0.7137	NA	0.89	Benign	NA
		SLC40A1	rs1439816	NA	Non-coding	Homozygous	NA	0.7967	0.8192	NA	0.96	Benign	NA

ACMG, American College of Medical Genetics and Genomics; ADPKD, Autosomal dominant polycystic kidney disease; AJ-MAF, Minor allele frequency in the Ashkenazi Jewish population; GD, Gaucher disease; MAF, Minor allele frequency; NA, Not available; REVEL, Rare exome variant ensemble learner; SIFT, Sorting intolerant from tolerant.

*Referred to as ASJ-MAF in common variant databases, including the gnomAD database.

**Unresolved—Does not meet criteria for blended phenotype; single heterozygous *NEB* VUS, in a recessive gene. Detailed data and rationale in Supplementary Case S5.

***Unresolved—Does not meet criteria for the blended phenotype; *NEB* synonymous VUS and heterozygous *ALPL* variant without functional validation. See Supplementary Case S6.

Below, we present selected cases to illustrate the GD phenotype, clinical reasoning process, therapeutic decision-making, and the diagnostic approach leading to the identification of multiple molecular diagnoses. These cases highlight the application of precision medicine in a single-gene disorder, demonstrating how individualized management was informed by deep phenotyping, longitudinal follow-up, and genomic insights.

### 3.1 Unusual inflammatory symptoms due to concurrent FMF

Patient 1: A 42-year-old man presented with mild splenomegaly, thrombocytopenia, and osteopenia and was diagnosed with GD1 due to homozygosity for the p.Asn409Ser *GBA1* mutation. He experienced recurrent severe serous pericarditis despite colchicine therapy, necessitating repeated hospitalizations and steroid treatment. Despite imiglucerase enzyme replacement therapy (ERT) for GD, recurrent pericarditis persisted, warranting a pericardiectomy. Pericarditis has been previously described in GD; hence, we expected an improvement in symptoms on ERT, but there was no improvement (Harvey et al., 1969; Benbassat et al., 1968). Episodes of recurrent pericarditis continued six or more times each year for more than 2 decades, requiring steroids. Finally, he transitioned to eliglustat substrate reduction therapy (SRT). His response to SRT was striking, with a sustained remission of pericarditis. The patient was able to discontinue colchicine, and there has been no recurrence of pericarditis for the past 6 years on SRT. WES, focusing on genes underlying inflammatory diseases, revealed a heterozygous p.Asp424Glu variant in the *MEFV* gene (The International FMF Consortium, 1997).

Patient 2: A woman presented at age 25 due to fatigue and bone pain. Genetic testing revealed homozygosity for the p.Asn409Ser *GBA1* mutation. Symptoms of fatigue, joint pain, and bone pain were debilitating. While the patient had elevated markers of GD, the severity of symptoms was disproportionate to the findings of GD markers. WES revealed a heterozygous p.Val726Ala variant in the *MEFV* gene previously reported to be associated with FMF (Aksentijevich et al., 1999). Initiation of eliglustat SRT and colchicine resulted in significant amelioration of symptoms.

Patient 3: An adult woman with a history of thrombocytopenia, easy bruising, and recurrent mucosal bleeding was diagnosed with GD1 at age 39 due to homozygosity for the p.Asn409Ser *GBA1* mutation. She developed hepatosplenomegaly, cytopenia, and osteopenia, along with debilitating ankle and abdominal pain. WES revealed a heterozygous p.Val726Ala variant in the *MEFV* gene. Her symptoms improved with eliglustat SRT and colchicine.

Patient 4: A 25-year-old woman presented with an unusual constellation of symptoms. The patient complained of abdominal distension, pain, nausea, malaise, polyarthralgia, and splenomegaly. An abdominal magnetic resonance imaging (MRI) revealed splenomegaly, while lab testing showed normal blood counts. She was subsequently diagnosed with GD based on low leucocyte acid β-glucosidase activity. Based on the severity of her symptoms, treatment was initiated with eliglustat SRT, which resulted in a reduction in GlcSph level to normal (<1 ng/mL), but the patient continued to complain of frequent episodes of fever, night sweats, persistent abdominal pain, and mouth sores. WES analysis revealed the p.Asp424Glu variant in the *MEFV* gene. The patient reported significant improvement in symptoms on a combination of eliglustat and colchicine.

### 3.2 Myopathy, osteoporosis, and chronic fatigue

While mild myopathy has been described in GD1, it is rarely severe enough to cause profound limb-girdle weakness or secondary osteoporosis (Tsai et al., 2008). Two additional patients (Table 3) exhibited neuromuscular phenotypes with variants of uncertain significance; both remain unresolved and are detailed in Supplementary Cases S5, S6.

### 3.3 Complex phenotypes arising from another rare genetic disease

In a subset of patients with GD, atypical phenotypes were observed that could not be fully explained by the *GBA1* genotype or the known clinical spectrum of GD. In these individuals, WES identified additional rare genetic variants consistent with second diagnoses. These concurrent conditions contributed to expanded or overlapping phenotypes that, in some cases, may have interacted with GD pathophysiology or complicated clinical management. These findings underscore the utility of WES in refining diagnoses, uncovering potential genetic contributors to phenotypic variability, and informing more individualized treatment strategies.

#### 3.3.1 Patient 5: metachromatic leukodystrophy (MLD)

A male child presented with increased tone and developmental regression in his first year of life. By 16 months, he had developed spasticity and ataxia with reduced mobility and loss of speech. Brain MRI showed diffuse white matter abnormality. MLD was diagnosed based on low leucocyte arylsulfatase A activity and abnormal urinary sulfatides. At age 4, he was noted to have thrombocytopenia and elevated liver enzymes. WES revealed compound heterozygous *GBA1* mutations, p.Asn409Ser and 84GinsG, consistent with GD1. Additionally, he was homozygous for the p.Arg60Trp variant in the *ARSA* gene that encodes arylsulfatase, known to cause late infantile MLD (Rabin et al., 2024). A recent study demonstrated a frequency of 1 in 1554 or 0.06% in the Ashkenazi Jewish population (Rabin et al., 2024).

The patient received ERT and supportive care. Unfortunately, he was not a candidate for a bone marrow transplant as his disease was deemed too advanced to offer meaningful benefits and unacceptable risks.

#### 3.3.2 Patients 6 and 7: fibromuscular dysplasia (FMD) and Brugada syndrome

Two patients in our cohort, homozygous for the *GBA1* p.Asn409Ser mutation, had cardiovascular disease. Cardiovascular complications are recognized in type 3c GD due to a homozygous p.Asp448His mutation (Wang et al., 2024). Cardiovascular disease is uncommon in GD1, with a lower prevalence of atherosclerotic complications compared to the general population, likely due to the typically low LDL cholesterol levels observed in these patients (Stein et al., 2011). However, in rare instances, GD1 patients may develop vascular or cardiac abnormalities, particularly when additional genetic factors are present. The following cases illustrate unique cardiovascular manifestations that occurred in two patients, highlighting the importance of considering the role of concurrent genetic conditions in shaping disease burden.

Patient 6: A 60-year-old woman presented at age 21 with massive splenomegaly and thrombocytopenia and was found to be compound heterozygous for the *GBA1* mutations p.Asn409Ser and p.Val433Leu. ERT with alglucerase (later imiglucerase) reversed her organomegaly and cytopenia. Subsequently, she transitioned to eliglustat SRT and maintained good control of her GD. At age 56, she had a spontaneous coronary artery dissection (SCAD) resulting in a non-ST elevation myocardial infarction (NSTEMI) with systolic cardiomyopathy. Cardiac catheterization showed increased tortuosity in distal coronary arteries without occlusion. Positron emission tomography (PET) and computed tomography angiography (CTA) imaging of the abdomen and pelvis revealed a beaded appearance in the right renal and external iliac arteries, suggesting FMD. WES revealed a c.724C>T (p.Gln242*) nonsense variant in the *YY1AP1* gene, previously implicated in Grange syndrome and FMD-like syndrome, which involves vascular abnormalities (Guo et al., 2017). While recognizing the possibility of this being a rare adverse effect of eliglustat, it is important to note that structural cardiovascular abnormalities have not been reported as a consequence of eliglustat SRT, and the patient was dosed pharmacologically with attention to drug–drug interactions, proven to ensure safety (Peterschmitt et al., 2019; Ain et al., 2025).

Patient 7: An adult man, who initially presented at age 14 with splenomegaly and thrombocytopenia, was diagnosed with GD1 due to homozygosity for the p.Asn409Ser *GBA1* mutation and commenced ERT. A routine EKG during screening for the eliglustat trial revealed a type I Brugada pattern in leads V1-V2. WES, focusing on genes associated with Brugada syndrome, revealed he was heterozygous for g.15206G>T splice acceptor variant in the *CACNB2* gene (Garcia-Elias and Benito, 2018). There were no variants in *SCN5A*, which is classically associated with Brugada syndrome; however, the *CACNB2* gene defects also result in Brugada syndrome (Garcia-Elias and Benito, 2018).

#### 3.3.3 Patients 8 and 9: constitutional mismatch repair deficiency (CMMRD) syndrome

Patients with GD have an increased risk of malignancies, particularly hematologic cancers, with epidemiologic studies suggesting an elevated incidence of multiple myeloma and lymphoma (Rosenbloom et al., 2022). However, these malignancies are predominantly observed in older adults, and cancer is rarely reported in pediatric GD patients. The following cases describe two siblings with GD1 who developed T-cell acute lymphoblastic lymphoma (T-ALL) at a young age, highlighting the potential contribution of an additional genetic predisposition.

Two siblings with GD1, born to non-consanguineous parents, developed T-ALL, previously reported by us (Lo et al., 2012). Both children presented with splenomegaly and mediastinal mass. Bone marrow aspirate revealed the presence of T-ALL as well as Gaucher cells. Exome analysis in these siblings revealed homozygosity for a novel GD mutation (p.Asp137Asn) in the *GBA1* gene and a homozygous c.3822dupA/c.3822dupA variant in the *MSH6* mismatch repair (MMR) gene (Lo et al., 2012).

### 3.4 ADPKD

Recognizing cases of GD with a family history of ADPKD is particularly important as dysregulation of GlcCer has been implicated in ADPKD pathogenesis. Studies suggest that SRT, including eliglustat, has been investigated as a potential therapeutic approach for ADPKD (Natoli et al., 2010). The following cases illustrate the coexistence of GD and ADPKD, underscoring the importance of considering the potential mechanistic interplay between lysosomal dysfunction and cystic kidney disease when managing these patients.

Patient 10: An adult woman with a known family history of polycystic kidney disease (PKD) was found to have hepatosplenomegaly and cytopenia at age 33. She was homozygous for the p.Asn409Ser mutation in the *GBA1* gene and was started on ERT. WES, focusing on genes associated with PKD, revealed heterozygosity for the p.Pro694Leu variant in the *PKD1* gene. Her GD has been well-controlled on ERT, and there has been no progression of her PKD, indicated by preserved renal function and no increase in renal cysts during 32 years of follow-up.

Patient 11: An adolescent male child was diagnosed with GD1 at the age of 2.5 years through family screening because his sibling was known to be affected. There was also a positive family history of PKD, and he was found to be affected by ultrasound. Exome analysis confirmed a diagnosis of ADPKD, revealing heterozygosity for the p.R4150C variant in the *PKD1* gene. During regular follow-up, he had up-trending indicators of GD activity and an increasing number of renal cysts, although his renal function was normal. At age 16, he was started on eliglustat SRT.

### 3.5 Myoclonic epilepsy

Epilepsy is a recognized neurological feature of GD3, often presenting as generalized or focal seizures (Tylki‐Szymańska et al., 2010). However, the devastating form of myoclonic epilepsy, which can significantly impact quality of life and disease progression, is uncommon in GD3 (Park et al., 2003). The following case describes a patient with GD3 who developed severe myoclonic epilepsy, highlighting the potential contribution of additional genetic factors to seizure susceptibility.

Patient 12: A 17-year-old woman was diagnosed with GD3 at the age of 20 months when she presented with pancytopenia, hepatosplenomegaly, developmental delay, and horizontal gaze palsy. A bone marrow biopsy revealed Gaucher cells, and subsequent investigations identified compound heterozygous *GBA1* mutations, p.Leu363Pro and Gly416Ser. Treatment was initiated with a combination of imiglucerase and miglustat. At age 16, she experienced subtle myoclonic jerks in her fingers, which evolved into episodes of generalized tonic-clonic seizures and myoclonic epilepsy requiring multiple anti-epileptic drugs. WES focusing on epilepsy genes revealed a heterozygous *EFHC1* variant, p.Arg294His, previously implicated in myoclonic epilepsy (Suzuki et al., 2004; de Nijs et al., 2012).

### 3.6 Hyperferritinemia and iron overload

Hyperferritinemia with normal iron saturation is a well-recognized feature of GD, often occurring as part of chronic inflammation rather than true iron overload (Motta et al., 2024). However, it is uncommon for GD patients to exhibit both elevated ferritin and iron saturation, with definitive evidence of iron overload on imaging and/or liver biopsy (Stein et al., 2010). The following cases illustrate rare instances where GD coexists with genetic predispositions to iron overload, highlighting the potential interplay between GD pathophysiology and iron metabolism.

Patient 13: Patient 13 is the same patient with GD and myopathy who is also described as Supplementary Case S5. She presented with splenomegaly, thrombocytopenia, and elevated ferritin at age 20. Initially diagnosed with chronic liver disease, her liver biopsy revealed Gaucher cells and hepatocyte siderosis. She was found to have low leukocyte acid β-glucosidase activity, and she was homozygous for the p.Asn409Ser *GBA1* mutation. Her symptoms improved on ERT. A routine MRI was performed to assess GD, which revealed iron overload in the liver and the bone marrow (Stein et al., 2010). She underwent phlebotomy to reverse iron overload. WES analysis revealed a homozygous p.His63Asp variant in the *HFE* gene (Yassin et al., 2014). Additionally, the patient exhibited homozygosity for both the c.44-24G>C and synonymous p.Val221= (alters the splice site) single-nucleotide polymorphisms (SNPs) in the *SLC40A1* gene, which have been linked to iron overload syndromes (Duca et al., 2022).

Patient 14: A male child presented with massive splenomegaly and avascular necrosis of the hips, and he was diagnosed with GD1. He underwent splenectomy at age 6 years and started ERT at age 23 years. He was homozygous for the p.Asn409Ser *GBA1* mutation. Later, he developed pain and swelling in his proximal interphalangeal joints and knee chondrocalcinosis. He was found to have hyperferritinemia and high iron saturation. MRI of the liver showed hepatic iron overload. WES revealed a heterozygous p.Cys282Tyr variant in the *HFE* gene. Additionally, the patient was homozygous for the c.44-24G>C and p.Val221 = SNPs in the *SLC40A1* gene (Duca et al., 2022). He is undergoing regular phlebotomies to manage the iron overload and recently switched to SRT due to persistent GD activity on ERT.

**FIGURE 2:**
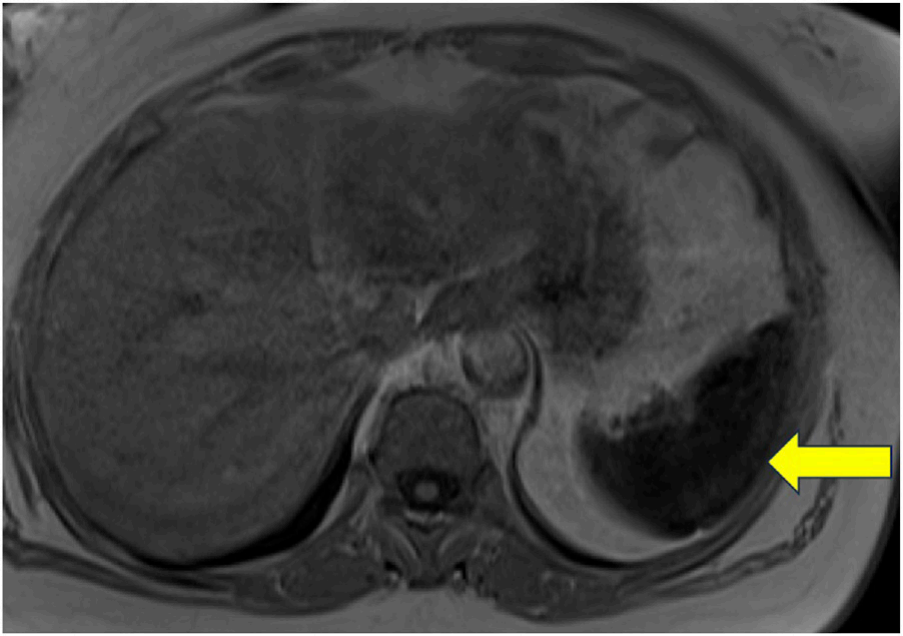
Non-contrast MRI of the abdomen of a patient with Gaucher disease and iron overload. This image represents a T1-weighted image with a uniformly hypointense spleen, which was associated with a signal dropout on the in-phase gradient echo sequence with a longer time to echo (TE), suggestive of diffuse iron deposition.

Patient 15: An adult woman of Italian ancestry presented at age 48 with thrombocytopenia and was diagnosed with GD1 due to a homozygous p.Asn409Ser *GBA1* mutation. Despite responding well to ERT, she had persistently elevated ferritin and splenic iron overload on MRI (Figure 2). WES revealed a heterozygous p.His63Asp variant in the *HFE* gene (Hanson et al., 2001). Additionally, she was homozygous for the c.44-24G>C and synonymous p.Val221 = SNPs in the ferroportin gene, *SLC40A1*.

Patient 16: A routine physical exam in an adult man at age 22 revealed elevated ferritin, thrombocytopenia, elevated liver function tests, and splenomegaly. Liver biopsy revealed hepatocyte siderosis as well as Gaucher cells. Leukocyte acid β-glucosidase activity was low, and he was found to be compound heterozygous for p.Asn409Ser and c.115 + 1G>A *GBA1* mutations. Despite a satisfactory response to ERT, he had persistent hyperferritinemia. Abdominal MRI revealed hepatic and splenic iron overload. WES revealed a heterozygous p.His63Asp *HFE* variant. He was also found to have the SNPs c.44-24G>C, UTR c.C98G, and UTR c.G8C in the *SLC40A1* gene. The latter two SNPs have been reported in Brazilian patients with primary iron overload (Santos et al., 2011).

Patient 17: Patient 17 was a male child who presented at age 48 with thrombocytopenia and hyperferritinemia and was diagnosed with GD1 due to a homozygous p.Asn409Ser *GBA1* mutation. He was started on ERT and later switched to eliglustat SRT. Despite a good response to therapy, he had persistent hyperferritinemia and marked splenic iron overload on MRI. WES revealed homozygosity for the c.44-24G>C and p.Val221 = variants in the *SLC40A1* gene.

## 4 Discussion

GD, though monogenic in origin, exhibits remarkable phenotypic heterogeneity that cannot be fully explained by the *GBA1* genotype alone. Our study expands this framework by demonstrating that, in approximately 6% of patients, atypical clinical features were attributable to multiple molecular diagnoses—supporting the growing recognition that multi-locus inheritance and blended phenotypes are not uncommon, with prior exome studies suggesting similar patterns in up to 5% of individuals undergoing genomic evaluation (Posey et al., 2017).

Building on prior reports showing that multiple molecular diagnoses can clarify atypical presentations and guide patient care, we applied a similar approach in GD (Malhotra et al., 2025; Kurolap et al., 2016). Importantly, this work moves beyond the enumeration of multiple molecular diagnoses to show how integrated genomic analysis can illuminate the underlying basis of unexplained clinical variation in GD. The accumulation of GlcCer and GlcSph in GD activates diverse downstream pathways, including inflammasome signaling, iron dysregulation, autophagy defects, and oxidative stress, which provide a plausible biological context for interaction with other rare disorders (Boddupalli et al., 2022; V et al., 2016).

Taken together, our findings highlight the value of combining deep phenotyping with genomic investigation to refine diagnoses, recognize potential clinically meaningful gene–gene interactions, and tailor therapeutic strategies. This integrated approach is particularly essential when clinical features deviate from the expected trajectory based on *GBA1* genotype or age at onset, reinforcing the role of precision medicine in optimizing care for patients with rare, multisystem diseases (Mistry et al., 2002). We focused on 17 patients with GD as a model to illustrate how precision medicine can be applied to rare monogenic disorders. By integrating deep phenotyping, longitudinal natural history, and genomic analysis, we were able to refine diagnoses and optimize therapeutic strategies in cases with atypical presentations. The classical phenotype of GD1 encompasses variable degrees of hepatosplenomegaly, cytopenia, and skeletal disease. However, in some individuals, concurrent genetic conditions appeared to amplify or distort these features in unexpected ways—prompting further investigation. For example, while hyperferritinemia is common in GD, iron overload, characterized by elevated iron saturation and hepatic iron deposition, is unusual and was observed in multiple patients with coexisting variants in iron metabolism genes (Stein et al., 2010). Similarly, although cancer risk is elevated in GD, pediatric malignancy is rare. The identification of homozygous *MSH6* variants in two siblings with GD and T-cell acute lymphoblastic lymphoma (T-ALL) highlights the importance of considering hereditary cancer predisposition syndromes in such contexts (Lo et al., 2012). Lastly, while mild muscle involvement is recognized in GD, the presentation of florid limb-girdle myopathy in one patient and disuse osteoporosis led to the identification of variants in the *NEB* gene associated with nemaline myopathy (Tsai et al., 2008).

In other cases, the presence of dual diagnoses was suspected *a priori* based on family history, as seen in patients with GD and ADPKD. Recognizing such co-occurrences is clinically important, particularly given emerging evidence linking GlcCer dysregulation to cystogenesis in ADPKD and the potential role of substrate reduction therapy (SRT) in modulating disease progression (Natoli et al., 2010). Similarly, while cardiovascular complications are typically confined to GD3c and are uncommon in GD1—likely due to the characteristically low LDL cholesterol levels—two GD1 patients in our cohort developed vascular and cardiac abnormalities associated with additional pathogenic variants in genes linked to fibromuscular dysplasia and Brugada syndrome, respectively (Wang et al., 2024; Stein et al., 2011). Finally, one patient was diagnosed with coexisting MLD and GD, which are two distinct lysosomal storage disorders. This case exemplifies how comprehensive phenotypic and genomic evaluation can reveal blended phenotypes and supports the rationale for including patients with atypical GD presentations in broader diagnostic frameworks.

### 4.1 Precision medicine in GD

This study outlines a practical framework for implementing precision medicine in the context of a single-gene disorder like GD. We identify three essential pillars that collectively support individualized care:

- Integrated clinical assessment—A comprehensive evaluation that combines clinical reasoning and longitudinal deep phenotyping. This approach uses long-term follow-up data, family history, phenotype, and disease trajectory to recognize when a patient’s presentation deviates from classical GD, prompting further genomic investigation.
- Genomic integration—Application of WES to uncover variants that explain atypical clinical features, refine diagnoses, and guide targeted interventions.
- Therapeutic individualization—Adaptation of treatment based on the presence of concurrent genetic conditions, such as using eliglustat in patients with inflammatory phenotypes or incorporating phlebotomy in cases of iron overload.

### 4.2 Genetic background and dual diagnoses

While some concurrent disorders may arise by chance, recurrent patterns across genetically defined populations suggest potential mechanistic or ancestral relevance. The co-occurrence of GD and FMF in individuals of Ashkenazi Jewish descent is particularly striking, given the high carrier frequency of both *GBA1* and *MEFV* variants in this population (Stirnemann et al., 2017; LIDAR et al., 2010). Historical genomic data from medieval Ashkenazi Jewish individuals have identified the presence of both the *GBA1* p.N370S and *MEFV* p.Val726Ala variants, suggesting a possible shared evolutionary background (Waldman et al., 2022).

Although heterozygous *MEFV* variants such as p.Val726Ala are typically associated with reduced penetrance, they may produce FMF-like symptoms in some carriers. Their potential role as modifiers of GD-related inflammation or disease expression remains a compelling but unresolved question (Booty et al., 2009; Berkun et al., 2012).

### 4.3 Pathway interactions and clinical implications

Finally, the convergence of GD with other genetic conditions in this cohort raises important questions about shared and intersecting biological pathways. In patients with GD and FMF, both disorders involve inflammasome activation, which may synergistically amplify systemic inflammation and upregulate GlcCer synthesis (Nair et al., 2015; Pandey and Grabowski, 2013; Pandey et al., 2017). This could explain the enhanced clinical response observed with glucosylceramide synthase (GCS) inhibitors like eliglustat compared to ERT. Similarly, in cases of GD with coexisting myopathy, chronic inflammation and metabolic stress may compound neuromuscular dysfunction, suggesting a mechanistic interaction between lysosomal and muscle structural pathways (González-Jamett et al., 2022). In patients with GD and iron overload, the unusual finding of hepatic iron deposition points to potential dysregulation of macrophage iron handling, possibly involving ferroportin-hepcidin signaling, an interaction not typically seen in isolated GD (Stein et al., 2010; Lefebvre et al., 2018). Finally, while seizures are known in GD3, the presence of disabling myoclonic epilepsy in one patient raises the possibility that additional genetic variants, such as those in *EFHC1*, may modify neuronal excitability and contribute to this rare phenotype (Thounaojam et al., 2017). A previous study implicated variants in the *SCARB2* gene in type 3 GD complicated by myoclonic epilepsy (Velayati et al., 2011). Together, these observations support a model in which concurrent genetic variants may modulate GD pathophysiology via shared immune, metabolic, or neuroinflammatory pathways, with implications for personalized therapeutic strategies.

### 4.4 Study limitations

Our study is limited by its single-center nature and the rarity of GD, which may limit generalizability. The heterogeneity of GD and varying depths of phenotyping across centers make replication difficult. In particular, variants of uncertain significance or single heterozygous variants in recessive disease genes, such as those observed in Patients 5 and 6, should not be over-interpreted as blended phenotypes; rather, they highlight the interpretive limits of current genomic analysis and the need for functional validation. Emerging evidence suggests that synonymous variants may still impact gene expression, mRNA stability, and translation efficiency (Kesner et al., 2023). WES excludes most non-coding regions, making it challenging to detect complex alleles. Additionally, WES is less reliable for identifying copy number variants (CNVs) or mosaicism due to its inherent limitations in read depth, coverage, and difficulty capturing structural variants.

While we describe only a small subset of our patients, these cases highlight a potential paradigm for precision medicine in GD. In some cases, the concurrent genetic disorders appear stochastic, for example, GD and myopathy or GD and iron overload. Genetic population structure may also play a role, as most of our patients are Ashkenazi Jewish. Thus, the co-occurrence of GD and FMF should be considered in atypical patients, especially in Ashkenazi Jewish patients, due to the ancestral relationship between *MEFV* and GD variants. However, stochastic co-occurrence is not confined to Ashkenazi Jewish patients; it can also be seen in non-Jewish patients, illustrated by the two children with GD and ALL.

While our cases suggest potential mechanistic links, they remain hypothesis-generating, necessitating larger multicenter studies to confirm our observations. Future research should leverage rare disease consortia (e.g., NORD Centers of Excellence) to validate these findings in broader populations.

## 5 Conclusion

This study reframes the search for a singular genetic modifier in GD, instead highlighting the intricate interplay of multiple genetic and phenotypic factors that shape disease expression. By integrating longitudinal deep phenotyping, rigorous clinical reasoning, and comprehensive genomic analysis, we demonstrate how precision medicine can be operationalized in the management of a rare monogenic disorder. This approach not only refines diagnosis and enhances therapeutic decision-making but also uncovers biologically plausible interactions between GD and coexisting genetic conditions. Our findings further suggest that standard GD therapies, especially SRT, may exert ancillary benefits on coexisting genetic disorders such as FMF, likely through shared biological pathways. As genomic technologies become increasingly accessible, this framework offers a path forward for individualized care in GD and provides a model for applying precision medicine across the broader landscape of rare diseases.

## Data Availability

The datasets presented in this article are not readily available because of ethical and privacy restrictions. Requests to access the datasets should be directed to the corresponding author at pramod.mistry@yale.edu.
