# Supplementary material for: Precision genomic profiling in Gaucher disease: insights from atypical presentations
Source: Front Genet. 2025 Nov 7;16:1553036. doi: 10.3389/fgene.2025.1553036 (PMC12634035; doi:10.3389/fgene.2025.1553036)
Supplement: Supplementary file 1 [file Table1.docx]

**Supplementary Table 1.** **Clinical characteristics of our cohort of patients with complex phenotype GD**

This table highlights the clinical characteristics of GD patients with complex phenotypes at our center (N=17)

| **Patient** | **Age**  **(Years)** | **GBA Genotype** | **Age at GD Diagnosis**  **(Years)** | **Concurrent Genetic Disorder** | **Age at Second Diagnosis**  **(Years)** | **Latest Treatment** | **Duration of Latest Treatment**  **(Years)** |
| --- | --- | --- | --- | --- | --- | --- | --- |
| Patient 1 | 64 | p.Asn409Ser/p.Asn409Ser | 42 | Familial Mediterranean Fever | 58 | SRT | 6 |
| Patient 2 | 41 | p.Asn409Ser/p.Asn409Ser | 25 | Familial Mediterranean Fever | 29 | SRT | 5 |
| Patient 3 | 50 | p.Asn409Ser/p.Asn409Ser | 39 | Familial Mediterranean Fever | 43 | SRT | 6 |
| Patient 4 | 26 | p.Asn409Ser/? | 25 | Familial Mediterranean Fever | 25 | SRT | 1 |
| Patient 5 | 12 | p.Asn409Ser/84GinsG | 4 | Metachromatic Leukodystrophy | 2 | SRT | 5 |
| Patient 6 | 61 | p.Asn409Ser/p.Asn409Ser | 21 | Fibromuscular Dysplasia | 59 | SRT | 14 |
| Patient 7 | 37 | p.Asn409Ser/p.Asn409Ser | 14 | Brugada Syndrome | 23 | ERT | 23 |
| Patient 8 | Deceased | p.Asp137Asn/p.Asp137Asn | 7 | Constitutional Mismatch Repair Deficiency | 9 | ERT | 3 |
| Patient 9 | 20 | p.Asp137Asn/p.Asp137Asn | 1 | Constitutional Mismatch Repair Deficiency | 4 | ERT | 16 |
| Patient 10 | 66 | p.Asn409Ser/p.Asn409Ser | 33 | Autosomal Dominant Polycystic Kidney Disease | 60 | ERT | 33 |
| Patient 11 | 17 | p.Asn409Ser/p.Asn409Ser | 16 | Autosomal Dominant Polycystic Kidney Disease | 12 | SRT | 1 |
| Patient 12 | 18 | p.Leu363Pro/p.Gly416Ser | 2 | Myoclonic Epilepsy | 12 | SRT | 2 |
| Patient 13 | 69 | p.Asn409Ser/p.Asn409Ser | 32 | Hemochromatosis | 32 | SRT | 9 |
| Patient 14 | 51 | p.Asn409Ser/p.Asn409Ser | 6 | Hemochromatosis | 47 | SRT | 1 |
| Patient 15 | 74 | p.Asn409Ser/p.Asn409Ser | 48 | Hemochromatosis | 73 | ERT | 26 |
| Patient 16 | 28 | p.Asn409Ser/c.115+1G>A | 22 | Hemochromatosis | 21 | ERT | 6 |
| Patient 17 | 66 | p.Asn409Ser/p.Asn409Ser | 48 | Hemochromatosis | 60 | SRT | 10 |

GD – Gaucher disease; ERT -Enzyme Replacement Therapy; SRT – Substrate Reduction Therapy
