# Supplementary material for: Precision genomic profiling in Gaucher disease: insights from atypical presentations
Source: Front Genet. 2025 Nov 7;16:1553036. doi: 10.3389/fgene.2025.1553036 (PMC12634035; doi:10.3389/fgene.2025.1553036)
Supplement: Supplementary file 2 [file DataSheet2.docx]

**Supplementary Cases**

*Supplementary Case 5*: An adult female presented at age 20 with splenomegaly, thrombocytopenia, and elevated ferritin. Initially diagnosed with hemochromatosis, liver biopsy revealed both hepatocyte siderosis and Gaucher cells. She was subsequently diagnosed with GD1 due to homozygosity for the p.Asn409Ser mutation. Although she initially responded well to ERT, she later developed progressive myalgia and exertional dyspnea. Cardiopulmonary exercise testing was suggestive of myopathy, and muscle biopsy demonstrated cytochrome c oxidase-deficient myofibers with preserved mitochondrial function. WES revealed a heterozygous p.Asp5398Asn variant in the *NEB* gene, which encodes nebulin, a structural protein associated with Nemaline myopathy when disrupted.^1,2^ This variant encodes for a missense mutation in the C-terminal region of nebulin, a domain that is highly conserved and essential for Z-disc assembly. Mutations clustered near the C-terminus may alter isoform diversity through abnormal splicing and are associated with nemaline myopathy.^3^

*Supplementary Case 6*: A 2-year-old male child presented with frequent fractures, low bone mass, proximal muscle weakness, and low serum alkaline phosphatase levels. He was homozygous for p.Asn409Ser mutation in *GBA1* gene. A right quadriceps biopsy revealed type II myofiber atrophy and cytoarchitectural changes consistent with myopathy. Some mobility was restored four years after beginning ERT; a reduction of GD biomarkers accompanied a decrease in the frequency of fractures and less bone pain. However, by 12-years-of age, his myopathic symptoms started to progress with florid limb-girdle myopathy. He was wheelchair-bound and had dyspnea due to diaphragmatic weakness. WES revealed a heterozygous synonymous variant (p.Asp8345=) in the NEB gene, reported as VUS in ClinVar.^4^ This synonymous variant lies among the last exons encoding the SH3 domain which is critical for nebulin’s interaction with other thin-filament proteins. The SH3 region is a hotspot for differential splicing and for nemaline-myopathy–associated mutations.^3^ There were no variants in other myopathy genes to explain this phenotype. Additionally, he was found to have a heterozygous variant, T273M, in *ALPL* gene, which is associated with autosomal recessive and autosomal dominant hypophosphatasia – it has been reported to have allele frequency of 0.21% in European Exome database.^5,6^

The carrier frequency of NEB-related nemaline myopathy among Ashkenazi Jews is approximately 1 in 108, leading to an estimated disease incidence of about 1 in 47,000; while that for GD is 1 in 15 carrier rate with estimated frequency of 1 in 850.

**References**

1. Lehtokari VL, Pelin K, Herczegfalvi A, et al. Nemaline myopathy caused by mutations in the nebulin gene may present as a distal myopathy. *Neuromuscular Disorders*. 2011;21(8):556-562. doi:10.1016/j.nmd.2011.05.012

2. Moreno CAM, Artilheiro MC, Fonseca ATQSM, et al. Clinical Manifestation of Nebulin-Associated Nemaline Myopathy. *Neurol Genet*. 2023;9(1). doi:10.1212/NXG.0000000000200056

3. Pelin K, Hilpelä P, Donner K, et al. Mutations in the nebulin gene associated with autosomal recessive nemaline myopathy. *Proceedings of the National Academy of Sciences*. 1999;96(5):2305-2310. doi:10.1073/pnas.96.5.2305

4. https://www.ncbi.nlm.nih.gov/clinvar/variation/95123/.

5. Dahir K, Nunes M. *Hypophosphatasia*. (Adam M, Feldman J, Mirza G, eds.). Gene Reviews; 2007. Accessed May 31, 2025. https://www.ncbi.nlm.nih.gov/books/NBK1150/

6. Tilden DR, Sheehan JH, Newman JH, et al. Phenotypic Profiling in Subjects Heterozygous for 1 of 2 Rare Variants in the Hypophosphatasia Gene (ALPL). *J Endocr Soc*. 2020;4(8). doi:10.1210/jendso/bvaa084
