# Supplementary material for: Precision genomic profiling in Gaucher disease: insights from atypical presentations
Source: Front Genet. 2025 Nov 7;16:1553036. doi: 10.3389/fgene.2025.1553036 (PMC12634035; doi:10.3389/fgene.2025.1553036)
Supplement: Supplementary file 3 [file DataSheet1.docx]

Supplement 1

High-Throughput Barcoded Sample Preparation, Sample Target Capture, and Data Analysis

Briefly, Genomic DNA was extracted from peripheral blood using the QIAGEN DNeasy Blood & Tissue Kit (Cat. No. / ID: 69504) according to the manufacturer's instructions. High-quality genomic DNA was confirmed by A260/A280 and A260/A230 ratios >1.8 using nanodrop and by confirming a single high molecular weight band on a 0.8% agarose gel electrophoresis. Fragmented DNA samples were then transferred to a 96-well plate, and library construction was completed using a liquid-handling robot. The captured fragments were PCR amplified and purified with AMPure XP beads. Samples were quantified by qRT-PCR using KAPA Biosystems kit, and the insert size distribution was determined with the LabChip GX. Prepared library concentrations were normalized to 2nM and loaded onto Illumina NovaSeq6000 S4 flow cells. Samples were sequenced using 101 bp paired-end sequencing reads according to Illumina protocols. The system's Real Time Analysis (RTA) software converted signal intensities to individual base calls during a run. Base calls were transferred from the machine's dedicated personal computer to the Yale High-Performance Computing cluster via a 1 Gigabit network mount at the Yale Center for Genome Analysis (YCGA) for downstream analysis.

Secondary analysis for enriched Exome libraries

The pipeline for secondary exome data analysis follows the GATK 4 best practices guidelines for generating called variants, with support for hg38 human reference.^1^ The analysis pipeline aligns the paired-end reads using BWA MEM to the hs38DH human reference, versions of the hg38 references that include decoy sequences.^2^ It then marks PCR duplicates using Picard’s Mark Duplicates command. The GATK 4 software is used to realign indels, recalibrate base quality scores, and generate Genomic Variant Call Format (GVCF) files for each sample.^3^ Once GVCF files have been generated, joint variant calling is performed, and variants are filtered using either hard filtering (if a small number of samples are involved) or variant quality score recalibration (if a whole cohort is being analyzed).

VCF files were annotated using the ANNOVAR and Variant Effect Predictor (VEP), as well as additional databases such as OMIM, GO and ClinVar, to capture (1) the prediction of the variants' functional effects (damaging nonsense, splice, missense protein-altering. or frameshift variants), (2) whether the variants are common or rare in the population, (3) the conservation/intolerance of the variant or the gene across related species and (4) any relationships between the genes/variants known phenotypes and the disease phenotype.^4–8^

**References**

1. Van der Auwera GA, Carneiro MO, Hartl C, et al. From FastQ Data to High‐Confidence Variant Calls: The Genome Analysis Toolkit Best Practices Pipeline. *Curr Protoc Bioinformatics*. 2013;43(1). doi:10.1002/0471250953.bi1110s43

2. Li H, Durbin R. Fast and accurate short read alignment with Burrows–Wheeler transform. *Bioinformatics*. 2009;25(14):1754-1760. doi:10.1093/bioinformatics/btp324

3. McKenna A, Hanna M, Banks E, et al. The Genome Analysis Toolkit: A MapReduce framework for analyzing next-generation DNA sequencing data. *Genome Res*. 2010;20(9):1297-1303. doi:10.1101/gr.107524.110

4. Wang K, Li M, Hakonarson H. ANNOVAR: functional annotation of genetic variants from high-throughput sequencing data. *Nucleic Acids Res*. 2010;38(16):e164-e164. doi:10.1093/nar/gkq603

5. McLaren W, Gil L, Hunt SE, et al. The Ensembl Variant Effect Predictor. *Genome Biol*. 2016;17(1):122. doi:10.1186/s13059-016-0974-4

6. https://www.omim.org/.

7. https://www.geneontology.org/.

8. https://www.ncbi.nlm.nih.gov/clinvar/.
